# Plant domestication decreases both constitutive and induced chemical defences by direct selection against defensive traits

**DOI:** 10.1038/s41598-018-31041-0

**Published:** 2018-08-23

**Authors:** Xoaquín Moreira, Luis Abdala-Roberts, Rieta Gols, Marta Francisco

**Affiliations:** 1 Misión Biológica de Galicia (MBG-CSIC), Apartado de correos 28, 36080 Pontevedra, Galicia Spain; 2 Departamento de Ecología Tropical, Campus de Ciencias Biológicas y Agropecuarias, Universidad Autónoma de Yucatán, Apartado Postal 4-116, Itzimná, 97000 Mérida, Yucatán Mexico; 3 Laboratory of Entomology, Wageningen University, PO Box 16, 6700 AA Wageningen, The Netherlands

## Abstract

Studies reporting domestication effects on plant defences have focused on constitutive, but not on induced defences. However, theory predicts a trade-off between constitutive (CD) and induced defences (ID), which intrinsically links both defensive strategies and argues for their joint consideration in plant domestications studies. We measured constitutive and induced glucosinolates in wild cabbage (*Brassica oleracea* ssp. oleracea) and two domesticated varieties (*B*. *oleracea* var. acephala and *B*. *oleracea* var. capitata) in which the leaves have been selected to grow larger. We also estimated leaf area (proxy of leaf size) to assess size-defence trade-offs and whether domestication effects on defences are indirect via selection for larger leaves. Both CD and ID were lower in domesticated than in wild cabbage and they were negatively correlated (i.e. traded off) in all of the cabbage lines studied. Reductions in CD were similar in magnitude for leaves and stems, and CD and leaf size were uncorrelated. We conclude that domestication of cabbage has reduced levels not only constitutive defences but also their inducibility, and that reductions in CD may span organs not targeted by breeding. This reduction in defences in domesticated cabbage is presumably the result of direct selection rather than indirect effects via trade-offs between size and defences.

## Introduction

The interaction between herbivorous insects and plants is one of the most common types of species interactions and over millions of years has shaped the evolution of plant defences^1,2^. These defences include a wide spectrum of both physical (e.g., trichomes, thorns, spines) and chemical traits (e.g., the production of secondary metabolites such as phenolics, glucosinolates, and alkaloids) that are toxic or deterrent to herbivores^3,4^. A key aspect in the study of plant defences is distinguishing between constitutive and induced levels of defensive traits, where the former refer to the basal levels of a defence trait and the latter refer to increased levels in response to herbivory^3,5,6^. Induced defences are thought to have evolved in environments where herbivory varies temporally^6^, whereas greater reliance on constitutive defences is thought to be beneficial in environments where herbivory is temporally consistent such that expressing high basal levels all the time is the optimal strategy^3^. The evolution of constitutive vs. induced defences can also be understood in relation to fitness costs and benefits associated with each strategy under different ecological contexts^4,7^. Induced defences are considered to be energetically cheaper than constitutive defences since the cost of producing them is realized only after damage occurs^6,8^. If these defensive strategies are costly, a trade-off may exist between constitutive and induced defences such that relative investment in each may vary along a continuum depending on the ecological context^9–11^.

Plant domestication conducted over several millennia has resulted in the modification of specific plant traits to enhance vegetative or reproductive growth (depending on the type of tissue or organ that is being selected), to increase nutrient content, or to improve taste for human consumption^12–15^. At the same time, however, selective breeding has frequently led to a reduction in levels of plant physical or chemical defences^13,16^, in many cases by direct selection because these traits are harmful or distasteful to humans and livestock. Alternatively, selection for larger organs or increased productivity has simply diluted defence levels in crop plants or reduced defences in cases where growth and defences trade off^13,17,18^. As a result of lowered defence levels, domesticated plants are generally more susceptible to pathogen infection and damage by phytophagous insects compared to their wild relatives^12,17–23^. However, most of the evidence supporting this pattern comes from studies measuring plant constitutive defences, ignoring the effects of domestication on defence inducibility and the potential interaction between constitutive and induced defences (but see Kempel *et al*.^9^ and Rowen & Kaplan^24^). Plants commonly employ both constitutive and inducible defences, which frequently concern the same trait. Expression of constitutive and induced defences are often not independent of each other. Consequently, studying these strategies in isolation may limit a full understanding of plant defence mechanisms^9^. In one of the few available examples, Kempel *et al*.^9^ found a trade-off between constitutive and induced defences in wild species but this condition was much weaker in cultivated species, suggesting that the evolutionary forces acting on trade-offs between defensive strategies in wild plant species have been disrupted during the domestication process. These results indicate that investigating the dual effects of domestication on induced and constitutive defences is necessary to fully understand how the domestication process has altered crop pest resistance and the evolution of plant defences under artificial selection.

Selection for higher yield and quality in domesticated plants may also have differential effects on plant defence allocation among various plant tissues (i.e., leaves, stems, roots), depending on which trait or plant tissue is the target of domestication^9,21,25^. One prediction is that domestication has led to greater reductions in plant defences in targeted (i.e., bred for use or consumption) than in non-targeted tissues because selection directly acts upon defensive traits found only in the targeted tissue^18^. However, studies comparing levels of defensive traits for crops and their wild relatives in targeted and non-targeted plant organs still remain scarce (but see Dicenta *et al*.^26^), therefore limiting our understanding on the specificity of domestication effects on plant defences and preventing generalizations about effects on plant defensive phenotypes.

Here we investigated whether domestication has affected plant constitutive chemical defences and their inducibility (i.e., ability to increase defences beyond constitutive levels in response to herbivore damage) using two (domesticated) varieties of *Brassica oleracea* (*B*. *oleracea* var. acephala and *B*. *oleracea* var. capitata) and their wild ancestor (*B*. *oleracea* spp. oleracea) (“plant lines” hereafter). Both varieties were domesticated and selected for increased leaf size. Plant species within the Brassicaceae family all produce glucosinolates, which are plant secondary metabolites that have been shown to confer resistance against insect herbivores^27,28^. Glucosinolates are present in all plant tissues and organs of *Brassica* plants, and are inducible in response to herbivory^29^. We first assessed the presence and strength of trade-offs (or, more broadly, negative co-variances) between size (measured in terms of leaf area) and investment in glucosinolate defences, and between inducible and constitutive concentrations of these defensive compounds to reveal potential mechanisms (i.e., direct or indirect via trade-offs) by which these defensive traits have been affected by domestication. We then investigated whether the strength of domestication effects on glucosinolate levels differed between plant tissues and tested the hypothesis that tissues targeted by domestication (leaves) exhibited a stronger reduction in defence chemistry relative to non-targeted tissues (stems). To address these goals, we measured leaf area (the trait targeted by domestication) in all three plant lines and quantified the concentrations of constitutive and induced levels of glucosinolates in leaves, as well as constitutive levels in stems (non-targeted tissue). Induced levels were achieved by exposing plants to feeding by caterpillars of the generalist leaf-chewing herbivore *Mamestra brassicae*, known to feed on both wild and cultivated cabbage.

## Results

### Effects of domestication on constitutive glucosinolate concentrations in leaves

**Figure 1:**
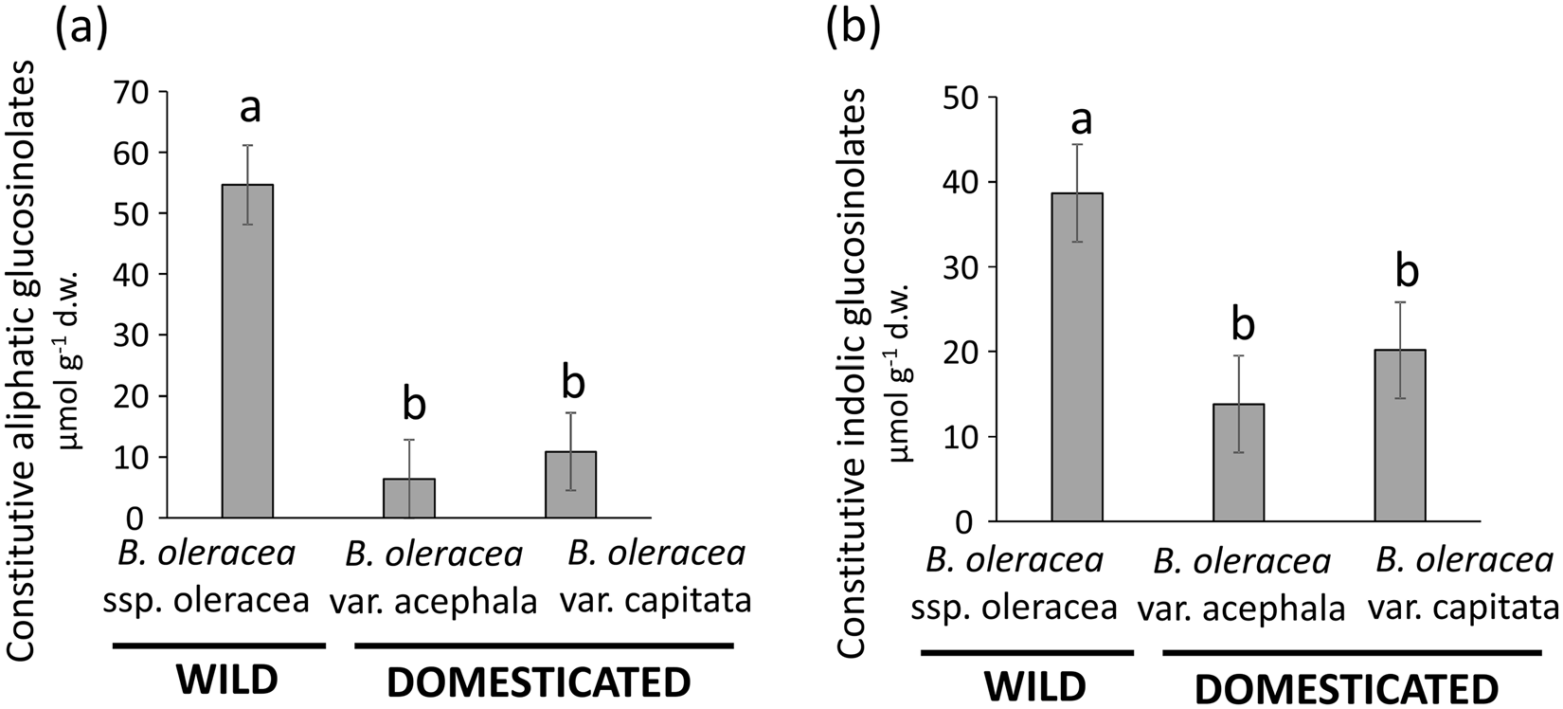
Effects of plant domestication on constitutive leaf chemical defences. Constitutive concentrations of **(a)** aliphatic glucosinolates and **(b)** indolic glucosinolates in leaves of wild cabbage (*Brassica oleracea* ssp. oleracea) and two domesticated varieties of this species (*B*. *oleracea* var. acephala and *B*. *oleracea* var. capitata). Bars are least square means ± s.e.m. (N = 60 plants per plant line). Different letters indicate significant (*P* < 0.05) differences between plant lines (see Table 1 for statistics). d.w. = dry weight.

**Figure 2:**
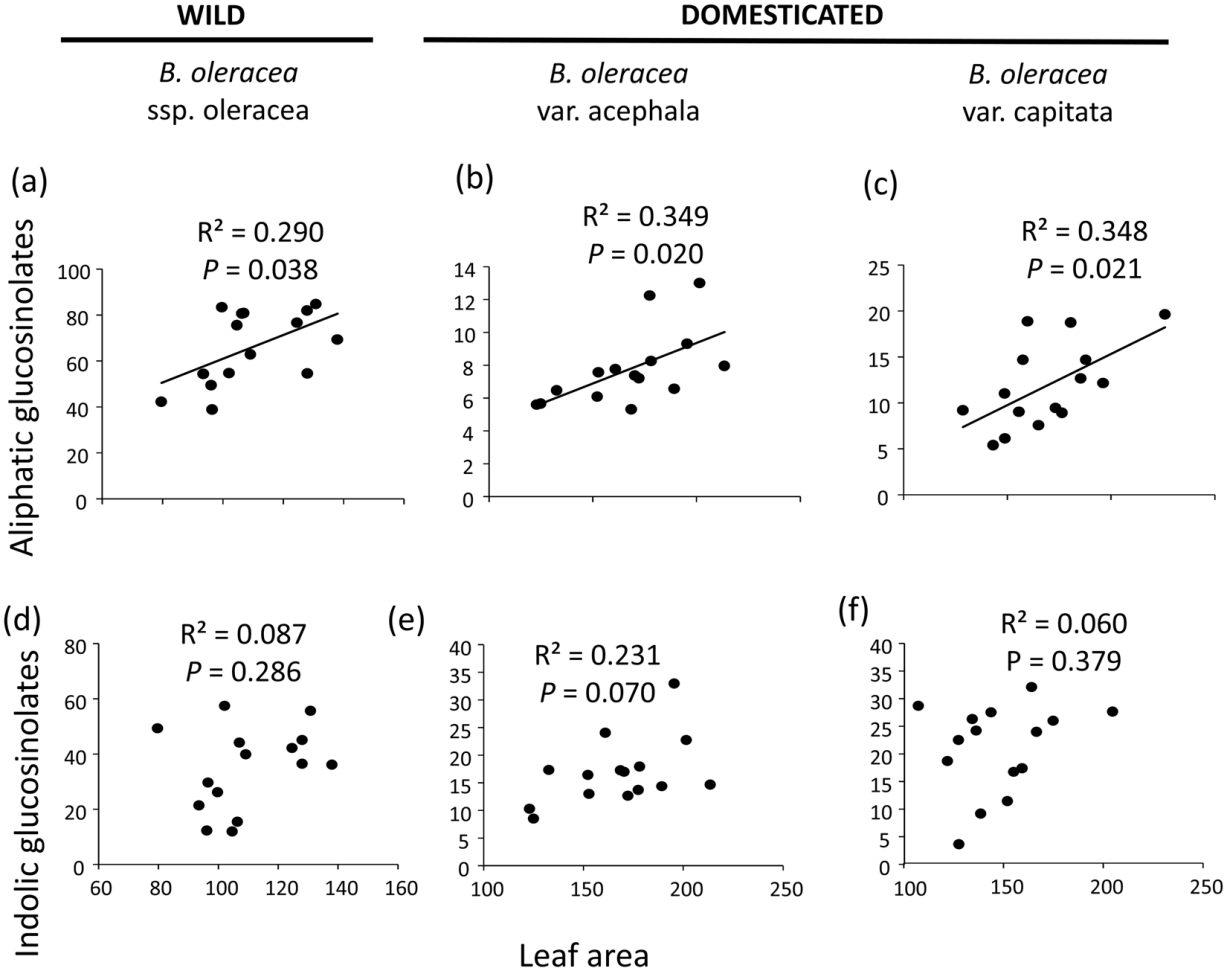
Correlations between constitutive glucosinolates and leaf area. Genetic correlations between leaf constitutive concentrations (in µmol g^−1^ d.w.) of (**a**–**c**) aliphatic glucosinolates and (**d**–**f**) indolic glucosinolates and leaf area (in cm^2^) in wild cabbage (*Brassica oleracea ssp*. *oleracea*) and two domesticated varieties of this species (*B*. *oleracea var*. *acephala* and *B*. *oleracea var*. *capitata*). R^2^ coefficients and associated *P*-values are shown. Each point represent a genotype (N = 15 genotypes).

Plant line had a significant effect on the concentration of both classes of glucosinolates in leaves (Table 1). Levels of constitutive aliphatic and indolic glucosinolates were significantly lower in *B*. *oleracea* var. acephala than in wild cabbage plants (Fig. 1a,b). Similarly, levels of constitutive aliphatic and indolic glucosinolates were significantly lower in *B*. *oleracea* var. capitata than in wild cabbage (Fig. 1a,b). Concentrations of both classes of glucosinolates did not differ significantly between the domesticated lines (Fig. 1a,b). There was also a significant positive relationship between leaf area and aliphatic glucosinolates (slope estimator = 0.0033 ± 0.0024, Fig. 2a–c), but a non-significant relationship between leaf area and indolic glucosinolates (Table 1, Fig. 2d–f), which together suggest that there were no trade-offs between leaf size and defensive investment. In addition, the interaction between plant line and leaf area was not significant for both classes of glucosinolates (Table 1), indicating that the relationship between leaf size and constitutive levels of glucosinolates was similar across plant lines.

**Table 1:** Results from general linear mixed models testing for the effects of plant domestication (i.e., plant line), leaf area and their interaction on constitutive levels of aliphatic and indolic glucosinolates in leaves.

Variable	DF	F-value	*P*
Constitutive aliphatics			
Plant line	2, 6	14.60	**0**.**005**
Leaf area	1, 120	12.36	**<0**.**001**
Plant line × leaf area	1, 120	0.11	0.894
Constitutive indolics			
Plant line	2, 6	7.30	**0**.**025**
Leaf area	1, 120	0.59	0.444
Plant line × leaf area	1, 120	1.24	0.293

We used three plant lines, the wild cabbage *Brassica oleracea* ssp. oleracea and two domesticated varieties of this species (*B*. *oleracea* var. acephala and *B*. *oleracea* var. capitata). We included leaf area (the tissue targeted for domestication) to test for a size-defence trade-off. The interaction term tests for different slopes of the relationship between constitutive glucosinolate levels and leaf area among the plant lines. F-values, degrees of freedom (numerator, denominator), and associated significance levels (*P*) are shown. Significant effects (*P* < 0.05) are in bold.

### Effects of domestication on the inducibility of glucosinolates in leaves

**Figure 3:**
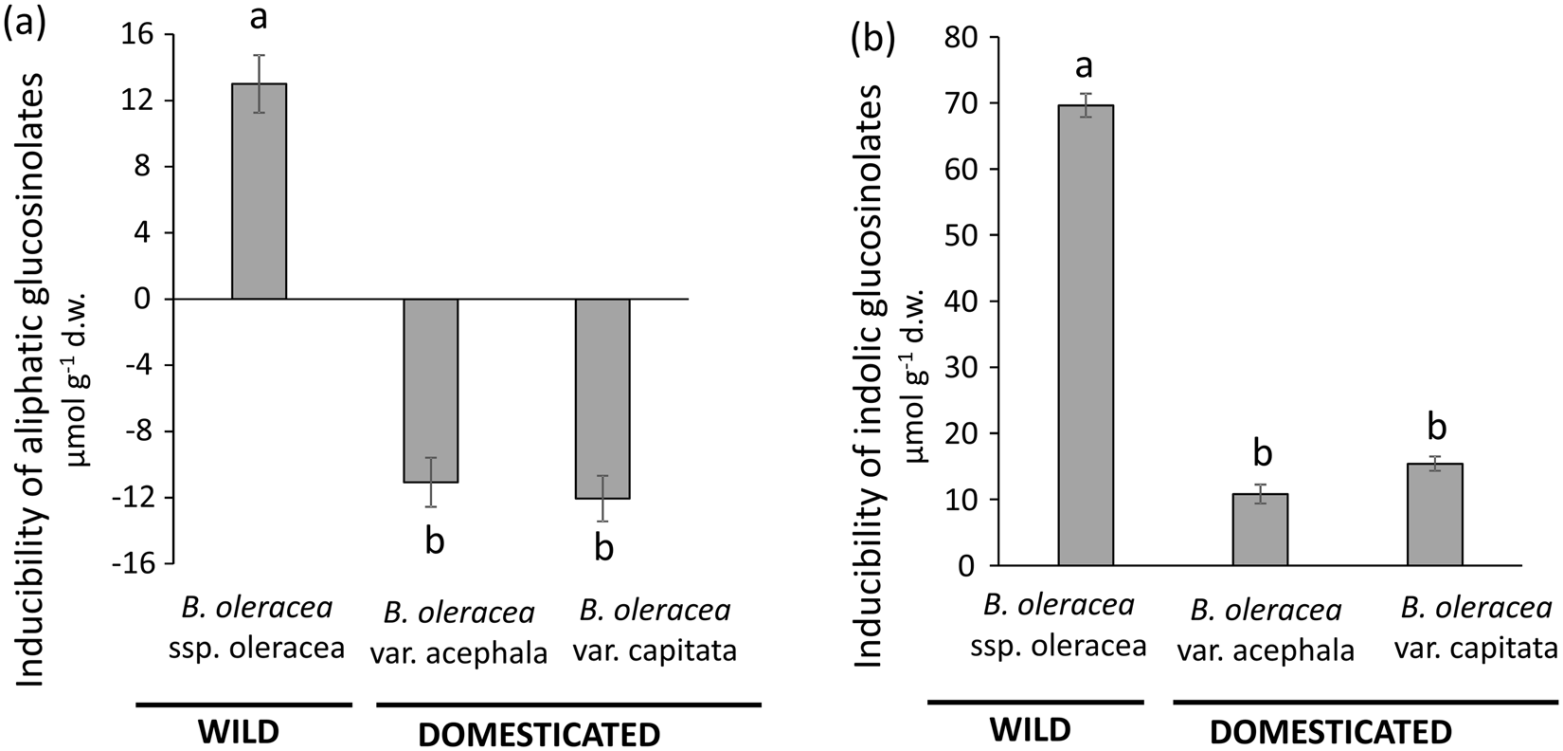
Effects of plant domestication on the inducibility of leaf chemical defences. Inducibility (i.e., the ability of plants to increase their defence levels in response to herbivory or some other type of stressor) of **(a)** aliphatic and **(b)** indolic glucosinolates in leaves of wild cabbage (*Brassica oleracea* ssp. oleracea) and two domesticated varieties of this species (*B*. *oleracea* var. acephala and *B*. *oleracea* var. capitata). Plants were induced by *Mamestra brassicae* larvae which fed on the plants for one week. Bars are least square means ± s.e.m. (N = 60 plants per plant line). Different letters indicate significant (*P* < 0.05) differences between plant lines (see Table 2 for statistics). d.w. = dry weight.

**Figure 4:**
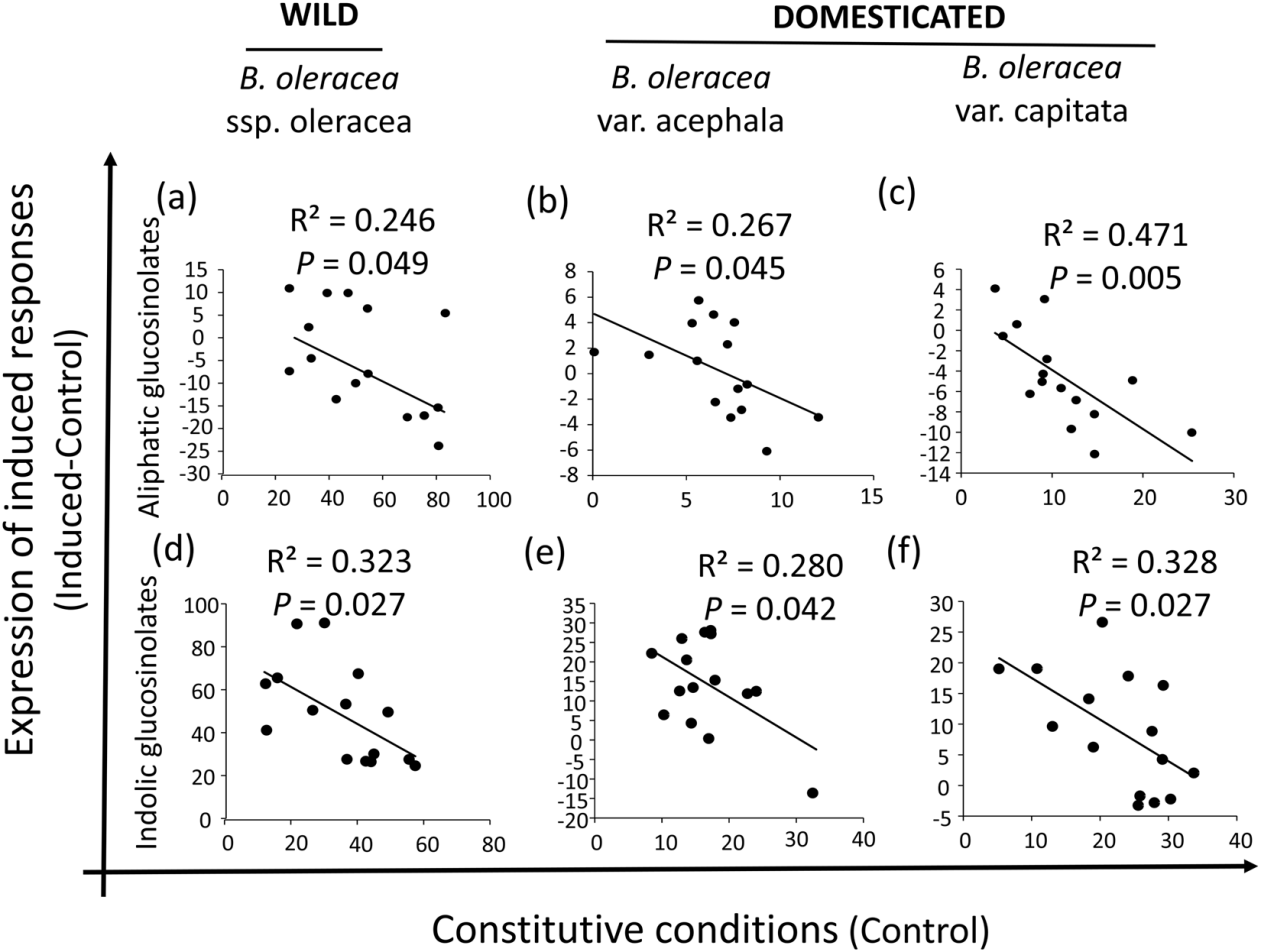
Correlations between constitutive glucosinolates and their inducibility. Genetic correlations between leaf constitutive concentrations (in µmol g^−1^ d.w.) of (**a**–**c**) aliphatic glucosinolates and (**d**–**f**) indolic glucosinolates and their corresponding inducibility by *Mamestra brassicae* damage in wild cabbage (*Brassica oleracea ssp*. *oleracea*) and two domesticated varieties of this species (*B*. *oleracea var*. *acephala* and *B*. *oleracea var*. *capitata*). Inducibility was measured as the genetic mean values in induced minus control plants. Negative significant correlations denote a genetic trade-off between constitutive and induced defences. R^2^ coefficients and *P*-values are shown. Each point represent a genotype (N = 15 genotypes).

There was a significant effect of plant line on inducibility of both classes of leaf glucosinolates (Table 2). In the case of aliphatic glucosinolates, we observed a significantly lower mean value of induced defences for both domesticated lines relative to the wild line, and no difference between domesticated lines (Fig. 3a). In fact, both domesticated lines exhibited induced susceptibility, i.e. a lower mean concentration of defences in induced than in control plants (Fig. 3a). In the case of indolic glucosinolates, all three lines had a significantly higher mean value of induced defences relative to controls (i.e., no evidence of induced susceptibility) with inducibility being significantly lower for both domesticated lines relative to the wild cabbage, and no difference between domesticated lines (Fig. 3b). In addition, we found a significant negative relationship between constitutive and inducible concentrations for both classes of glucosinolates (aliphatics: slope estimator = −0.635 ± 0.042; indolics: estimator = −0.617 ± 0.103, Table 2, Fig. 4a–f), suggestive of trade-offs between these defence strategies. However, for both classes of glucosinolates the interaction between plant line and constitutive levels was not significant (Table 2), i.e. the strength of the trade-off was similar among all plant lines regardless of their domestication status. Finally, herbivore damage (weighted by leaf area) had a significant positive effect on the inducibility of indolic glucosinolates (slope estimator = 941.70 ± 115.81), but a non-significant effect on the inducibility of aliphatic glucosinolates (Table 2).

**Table 2:** Results from general linear mixed models testing for the effects of plant domestication (i.e., plant line) on the inducibility of aliphatic and indolic glucosinolates in leaves.

Variable	DF	F-value	*P*
Inducibility of aliphatics			
Plant line	2, 28	24.93	**<0**.**001**
Constitutive aliphatics	1, 588	115.55	**<0**.**001**
Plant line × constitutive aliphatics	2, 492	2.99	0.052
Damage	1, 641	2.44	0.119
Inducibility of indolics			
Plant line	2, 9	49.62	**<0**.**001**
Constitutive indolics	1, 33	26.07	**<0**.**001**
Plant line × constitutive indolics	2, 8	0.11	0.899
Damage	1, 640	66.12	**<0**.**001**

We used three plant lines, the wild cabbage *Brassica oleracea* ssp. oleracea and two domesticated varieties of this species (*B*. *oleracea* var. acephala and *B*. *oleracea* var. capitata). We included constitutive defences in each case to test for a trade-off between constitutive and induced levels for each group of compounds. The interaction term tests for a difference between plant lines in the slope of the relationship between constitutive and inducible defences (i.e., constitutive-induced defence trade-offs). We also included leaf damage score weighted by leaf area as a covariate to account for variation in the amount of damage inflicted on plants from the herbivore-induced treatment. F-values, degrees of freedom (numerator, denominator) and associated significance levels (*P*) are shown. Significant effects (*P* < 0.05) are in bold.

### Effects of domestication on leaf vs. stem constitutive glucosinolates

**Figure 5:**
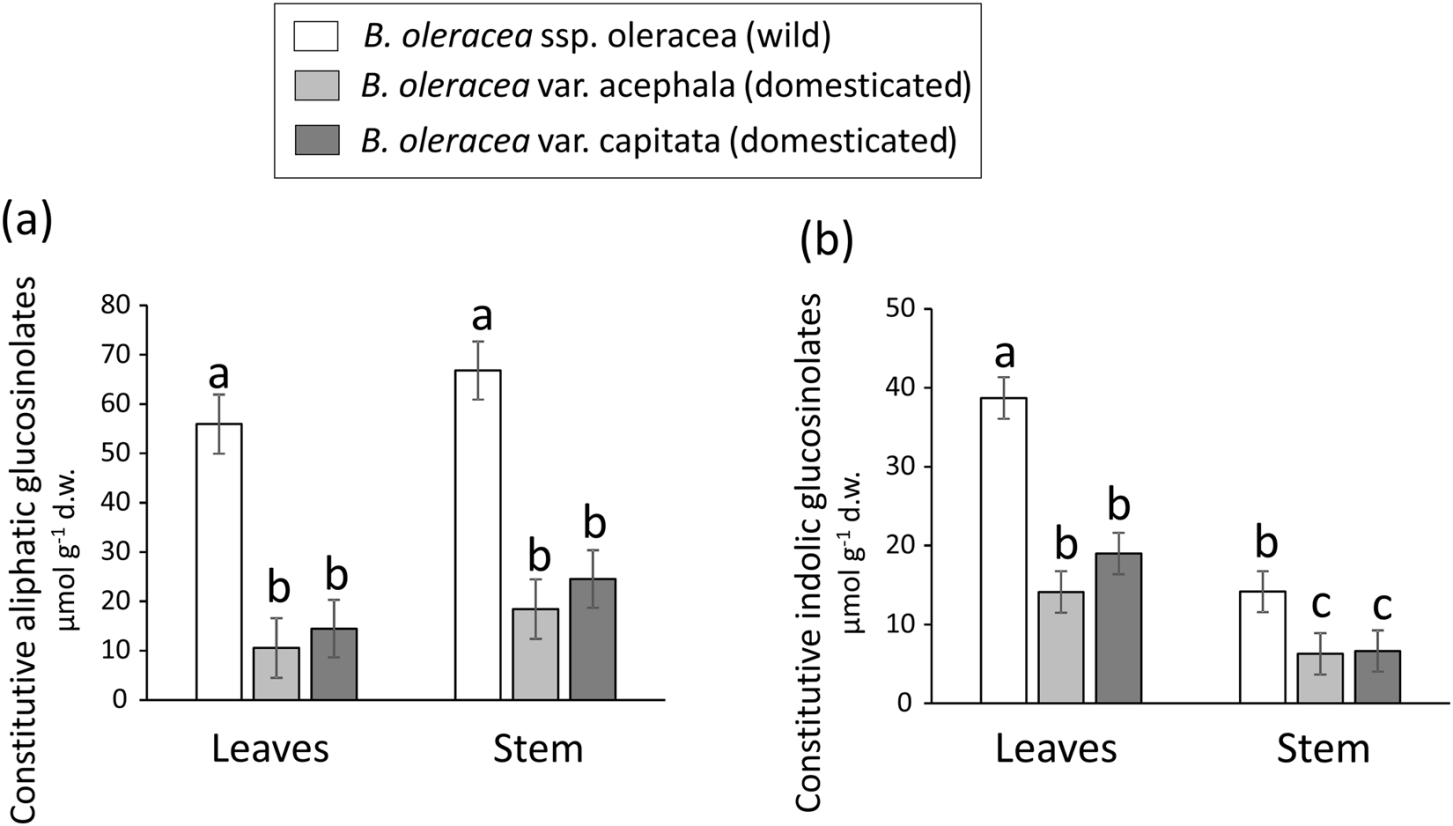
Effects of plant domestication on leaf vs. stem constitutive chemical defences. Constitutive concentrations of **(a)** aliphatic and **(b)** indolic glucosinolates in leaves and stems of wild cabbage (*Brassica oleracea* ssp. oleracea) and two domesticated varieties of this species (*B*. *oleracea* var. acephala and *B*. *oleracea* var. capitata). Bars are least square means ± s.e.m. (N = 36 replicate plants per plant line). Different letters indicate significant (*P* < 0.05) differences between plant lines (see Table 3 for statistics). d.w. = dry weight.

There were significant effects of plant line and tissue type on constitutive levels of aliphatic and indolic glucosinolates (Table 3). As above, the concentration of both classes of compounds was lower in the domesticated lines than in wild cabbage (Fig. 5a,b). In addition, constitutive levels of indolic glucosinolates were higher in leaves than in stems, whereas concentrations of aliphatic glucosinolates were higher in stems (the non-targeted tissue) than in leaves (Fig. 5a,b). Counter to expectations, the plant line by tissue interaction was not significant for either class of compounds (Table 3), indicating that in both cases the magnitude of difference in constitutive defences among plant lines was similar for both types of tissue (Fig. 5a,b).

**Table 3:** Results from general linear mixed models testing for the effects of plant domestication (i.e., plant line), plant tissue (leaves vs. stem), and their interaction on constitutive levels of aliphatic and indolic glucosinolates in leaves and stems.

Variable	DF	F-value	*P*
Constitutive aliphatics			
Plant line	2, 6	51.87	**<0**.**001**
Tissue	1, 162	36.73	**<0**.**001**
Line × Tissue	2, 162	2.16	0.119
Constitutive indolics			
Plant line	2, 6	26.50	**<0**.**001**
Tissue	1, 162	160.21	**<0**.**001**
Line × Tissue	2, 162	1.48	0.230

We used three plant lines, the wild cabbage *Brassica oleracea* ssp. oleracea and two domesticated varieties of this species (*B*. *oleracea* var. acephala and *B*. *oleracea* var. capitata). F-values, degrees of freedom (numerator, denominator), and associated significance levels (*P*) are shown. Significant effects (*P* < 0.05) are in bold.

## Discussion

Our results show that constitutive levels of glucosinolates as well as their inducibility were lower in domesticated than in wild *B*. *oleracea*. In addition, we found that inducible and constitutive levels of aliphatic and indolic glucosinolates were negatively correlated but this relationship was similar in strength in both domesticated and wild cabbage, suggesting that domestication has not influenced this defensive trade-off. Surprisingly, leaf size and constitutive concentrations of glucosinolates were either not correlated (in the case of indolic glucosinolates) or were weakly positively (alipahtic glucosinolates) correlated in all three cabbage lines studied. This result provides evidence that there is no indirect negative effect of domestication on constitutive defences due to selection for larger leaves (i.e., via growth-defence trade-offs). Finally, our results did not support the prediction that selection for reduced constitutive glucosinolate levels would primarily affect leaves, the tissue targeted by domestication, and thus lead to greater differences in defence levels for domesticated vs. wild cabbage in the case of leaves than in stems. Instead, the magnitude of difference between glucosinolate concentrations in leaves and stems was similar in domesticated and wild cabbage.

Constitutive concentrations of glucosinolates in leaves of *B*. *olereacea* were consistently lower in domesticated lines than in wild cabbage. These findings are in agreement with previous studies reporting reduced defences in domesticated crops relative to their wild relatives^17,18,20,30^. Two mechanisms have been proposed to explain this pattern. First, domestication may directly select for lower concentrations of compounds that are toxic, distasteful or have anti-nutritive properties for humans or livestock^18^. Alternatively, domestication may indirectly reduce plant chemical defences by selecting for increased quality or size (of targeted tissues), either through “dilution” of defensive compounds in enlarged organs or due to resource allocation constraints where increased growth results in a concomitant reduction in defence investment (i.e., growth-defence trade-offs^18^). We found no evidence for a trade-off between leaf size and defence investment, a pre-requisite for indirect effects of domestication. This finding therefore leaves direct selection for reduced glucosinolate concentrations, independently of selection of enlargement of edible structures, as the most probable mechanism of domestication effects on defences for the studied cabbage varieties. Consistent with our findings, a recent study by Turcotte *et al*.^17^ did not find support either for the predicted trade-off between growth and defence using 29 pairs of crops and their wild relatives. Similarly, Kempel *et al*.^9^ found no trade-offs between growth rate and constitutive resistance in 18 wild plant species and 40 cultivated garden-plant species. Overall, these findings and our results for cabbage suggest that indirect effects of domestication on plant defences via growth- or size-defence trade-offs may be less important than previously thought.

Notably, we found that domesticated lines of *B*. *oleracea* exhibited lower inducibility of both aliphatic and indolic glucosinolates compared to wild cabbage, but that the two classes of glucosinolate compounds exhibited qualitative differences in their induction patterns. In the domesticated lines, concentrations of aliphatic glucosinolates were lower in herbivore-induced than in control plants which is evidence of induced susceptibility, whereas for indolic glucosinolates concentrations were consistently higher in induced than in control plants. These results agree with previous work reporting that indolic glucosinolates generally increase in response to herbivory, whereas changes in aliphatic glucosinolates after damage are more idiosyncratic^29^. In relation to these findings, previous work has shown that inducibility of chemical defences depends on plant ontogeny or phenological stage. For example, Massei & Hartley^31^ found that ungulate browsing induced an increase in the phenolic content of wild and domesticated olives (*Olea europaea*) in winter but not in spring, and Chen & Romena^32^ observed higher survival of larvae of the yellow stem borer (*Scirpophaga incertulas*) on cultivated accessions of rice than on wild ones when plants were at the reproductive stage but not at the vegetative stage. Based on these findings, it would be worthwhile to further investigate whether observed patterns for inducibility of aliphatic and indolic glucosinolates in cabbage exhibit ontogenetic or phenological changes. This would produce novel information on whether domestication has influenced ontogenetic trajectories or phenological shifts in plant defence investment.

Importantly, results indicated a negative correlation between induced and constitutive levels for both indolic and aliphatic glucosinolates, suggesting a trade-off between these defensive strategies in both cases. However, the association between constitutive and inducible glucosinolates was statistically indistinguishable between wild and domesticated plant lines, suggesting that domestication in *Brassica* has not influenced trade-offs between induced and constitutive defences. In contrast, Kempel *et al*.^9^ found that this defensive trade-off was apparently stronger in wild that in cultivated species, but their study included mostly ornamental plants rather than vegetable crops which may lead to different patterns due to differences in the domestication process. We cannot, however, reject the possibility that inducibility of glucosinolates trades off with other defensive traits or mechanisms^6^. Follow-up work measuring inducibility of physical defences (e.g., toughness, trichomes) or tolerance mechanisms (e.g., compensatory growth and reproduction) in wild and domesticated cabbage would provide a more integral assessment of domestication effects on *Brassica* defences. Likewise, including a greater number of wild populations and cultivated accessions spanning a broader geographical distribution would be desirable to achieve a more robust evaluation of constitutive-induced trade-offs in this species and the mechanisms of domestication effects on cabbage defences.

It has been hypothesized that artificial selection for increased palatability and reduced toxicity of desired plant tissues or organs should primarily influence tissues targeted by breeding, whereas defences should exhibit no or little change in non-targeted tissues^18,21^. Counter to this expectation, we found that the magnitude of reduction in constitutive levels of glucosinolates was not contingent upon the plant tissue under study (i.e., non-significant interaction between tissue type and plant line). In other words, despite targeted selection to increase leaf size and/or reduce toxicity in *B*. *oleracea*, the magnitude of reduction in these constitutive defences between domesticated and wild plants was similar for both types of plant tissues. The results of the present study suggest that the biosynthesis of glucosinolates in leaves and stems is genetically correlated^33^. Glucosinolates are found in all plant organs of brassicaceous plants and different organs have been targeted for domestication in *Brassica* crops. It would be worthwhile to investigate how glucosinolate levels in different plant organs are affected in *Brassica* crops for which different organs are harvested to have a more complete understanding of domestication effects on within-plant glucosinolate defence allocation patterns.

Overall, the domesticated lines of *B*. *oleracea* in this study have undergone substantial reductions in constitutive defences and this effect has been of similar magnitude in both targeted and non-targeted tissues. Such effects have most likely been the result of direct selection to reduce defences rather than indirectly via correlated effects on plant growth or organ size. Furthermore, our findings show that domestication of cabbage for leaf consumption has led to strong reductions in the inducibility of glucosinolates. Although results indicate that inducible and constitutive levels of glucosinolates traded off, this compromise between defensive functions has apparently not been affected by domestication. Overall, these findings attest the importance of assessing the dual effects of domestication on plant constitutive and induced defences as well as the consistency of such effects across plant tissues in order to achieve a more comprehensive understanding of how artificial selection has influenced complex plant defensive phenotypes and the mechanisms underlying reduced insect pest resistance in domesticated plants.

## Material and Methods

### Natural history

Wild cabbage, *B*. *oleracea* L. ssp. oleracea, grows along the Atlantic coast of Europe. It is perennial and produces only vegetative tissues during its first year of growth and may produce flowers in the second year depending on environmental conditions. Artificial selection on leaves, terminal buds, lateral buds, stems and inflorescences has resulted in a large degree of phenotypic diversity among the different varieties of *B*. *oleracea*^34^. Particularly, in the case of *B*. *oleracea* var. acephala domestication has increased the size of the leaves relative to wild *B*. *oleracea* for purposes of human and animal consumption^35^. In turn, this leafy variety is considered the progenitor of other varieties such as *B*. *oleracea* var. capitata, for which selection was aimed at retaining the large leaves but also focused on dramatically reducing the internode length and enlarging the size of the terminal bud^35^.

Wild and domesticated cabbage plants are attacked by a large community of specialist and generalist insect herbivores, mainly leaf chewers (e.g., larval stages of Lepidoptera) and sap-feeders (e.g., aphids and white flies^36–39^). Among these herbivores, the generalist cabbage moth (*Mamestra brassicae*, Lepidoptera) is one of the most destructive pests attacking more than 70 plant species from 22 families, including wild and domesticated cabbage plants which are attacked at all growth stages^37,38,40^. Larvae of this species feed at night on the underside of the external leaves where they make small perforations and cause extensive damage^38^.

### Seed sources

Seeds of wild cabbage were collected from five individual plants (i.e., maternal families composed of half-sibs) originating from three populations located within a range of 15 km on the southern coast of the United Kingdom (Dorset, England). These populations are located at Durdle Door (50°62′N, 2°27′W), St. Aldhelms Head (50°69′N, 2°05′W), and Old Harry (OH; 50°38′N, 1°55′W). Previous work with these populations has shown that they are genetically sub-structured and gene flow among them is low but significant^41^. For each domesticated variety, we used seeds from five accessions (Table SM1 in the Supplementary Material, germplasm collection of the Biological Mission of Galicia, CSIC, Spain) and each quintet of accessions belonged to one of three distinct “regions” in Galicia separated by 2 to 270 km (north-western Spain, see Table SM1 in the Supplementary Material). We randomly chose seeds of wild and domesticated lines.

### Experimental design

The experiment followed a randomized split-plot design replicated over four blocks, where the two herbivore treatments were applied at the whole-plot factor and plant line was the split factor (Fig. SM1 in the Supplementary Material). We randomly assigned the 15 genotypes/accessions of both wild and domesticated lines within each split factor (Fig. SM1 in the Supplementary Material). In total, we grew 360 cabbage plants, corresponding to 4 blocks × 3 plant lines × 3 populations/regions × 5 genotypes × 2 treatments (control and exposed to *M*. *brassicae* larvae).

In January 2017, we grew all plants individually from seeds sowed in 4-L pots containing potting soil with peat. Plants were grown in a glasshouse under controlled light conditions (minimum 12 h per day) and temperature (10 °C night, 25 °C day), and were watered daily. Two months after germination, we measured total height and counted the number of leaves and estimated the mean leaf area by averaging the surface area of three randomly chosen leaves of a plant. To calculate leaf surface area, we used the formula for the area of an ellipse: π.A.B, where A and B are the maximum width and length of a leaf, respectively^42^. As expected, mean leaf area was significantly greater in domesticated cabbages than in their wild counterpart (F_2,6_ = 10.76, *P* = 0.010; *B*. *oleracea* var. acephala: 159.9 ± 9.8 cm^2^, *B*. *oleracea* var. capitata: 170.3 ± 9.7 cm^2^, *B*. *oleracea* ssp. oleracea: 106.9 ± 9.7 cm^2^).

Immediately after initial plant measurements, we performed the two herbivore treatments: (1) exposed to *M*. *brassicae* larvae (“induced treatment” hereafter) and (2) non-exposed to *M*. *brassicae* larvae (“control treatment” hereafter). For the induced treatment, three third-instar larvae were placed on three fully expanded leaves. To avoid treatment cross-contamination due to plant aerial communication via volatiles, control and induced plants were placed in separate greenhouse chambers at the time of induction and remained separated for 48 h after induction. Before the larvae of *M*. *brassicae* were placed on the plants, they were reared on a wheat germ-based artificial diet. The larvae were allowed to feed on the plants for one week and then were removed. At this point, we measured plant height, estimated the amount of damage (for herbivore-induced plants), and harvested leaf and stem tissues which were stored in a freezer at −80 °C for subsequent chemical analyses. We estimated herbivore damage levels on each of the three damaged leaves by using a six-level scale (0 = undamaged; 1 = 1–5% damaged; 2 = 6–10% damaged; 3 = 11–15% damaged; 4 = 16–20% damaged; 5 = > 20% damaged). We found few plants with more than three damaged leaves, indicating that *M*. *brassicae* larvae rarely moved among leaves.

### Chemical analyses

One week after establishing the herbivore treatments, we collected three randomly chosen leaves of each plant in the control group and the three herbivore-damaged leaves of all induced plants. We also collected samples of the stem of control plants in a subset of nine genotypes per plant line (3 plant lines × 9 genotypes × 4 blocks = 108 plants). All samples were freeze dried (BETA 2–8 LD plus, Christ) for 72 h and subsequently pulverized using an IKA-A10 (IKA-Werke GmbH & Co.KG) mill.

We extracted glucosinolates according to a previously described high-throughput analytical method^43,44^. Briefly, we weighed ten mg of each freeze-dried and pulverized sample in a single well of 96-well microliter plate containing 400 µL of 90% methanol and one 3.8 mm stainless steel ball-bearing. We homogenized tissues for 3 min, centrifuged them, and transferred the supernatants to a 96-well filter plate with 50 µL of DEAE sephadex. We eluted the sephadex-bound glucosinolates with 110 µL of sulfatase following overnight incubation at room temperature. We used ten µL of the desulfo-glucosinolates extract to identify and quantify the glucosinolate compounds. We carried out the chromatographic analyses on an Ultra-High-Performance Liquid-Chromatograph (UHPLC Nexera LC-30AD; Shimadzu) equipped with a Nexera SIL-30AC injector and one SPD-M20A UV/VIS photodiode array detector. The UHPLC column was an XSelect HSS T3 XP ColumnC18 protected with a C18 guard cartridge. The oven temperature was set at 30 °C. Compounds were separated using the following method in aqueous acetonitrile, with a flow of 0.5 mL min−1: 1.5 min at 100% H_2_O, an 11 min gradient from 0% to 25% (v/v) acetonitrile, 1.5 min at 25% (v/v) acetonitrile, a minute gradient from 25% to 0% (v/v) acetonitrile, and a final 3 min at 100% H_2_O. We recorded the data on a computer with the LabSolutions software (Shimadzu). We quantified all glucosinolate at 229 nm by using glucotropaolin (GTP, monohydrate from Phytoplan, Diehm & Neuberger GmbH, Heidelberg, Germany) as internal standard and quantified them by comparison to purified standards of progoitrin, gluconapin, glucoraphanin, sinigrin and glucobrassicin. We reported the concentration of glucosinolate compounds (aliphatic and indolic glucosinolates, two classes of glucosinolates based on the amino acid from which they have derived) as μmol g^−1^ dry weight (d.w.).

### Statistical analyses

We tested for an effect of domestication on constitutive defences by running general linear mixed models separately for constitutive levels of aliphatic and indolic glucosinolates using only control (undamaged) plants. In each model, we entered concentration of the phytochemical class as a response variable and plant line (three levels, two domesticated and one wild), leaf area, and their interaction as explanatory variables. In addition, block, population/region nested within plant line, and maternal line/accession nested within population/region were included as random factors (Fig. SM1 in the Supplementary Material). Leaf area was included to test for an association between investment in leaf size and constitutive defences, where a negative association suggests a trade-off between leaf size and defence investment. In addition, the interaction between plant line and leaf area tests for differences among plant lines in the slope of the linear relationship between constitutive defences and leaf area (whether the trade-off differs among plant lines). Additionally, to test for trade-offs between plant size and constitutive defences, we also performed genetic correlations (using genotypic means) between constitutive glucosinolates and leaf area. We preferred to treat both accessions and wild cabbage as individual levels within the “plant line” factor rather than test for an overall effect of status (e.g., by coding both varieties as domesticated) because it allowed us to explicitly evaluate the effects of each domestication event (one for each variety) by separately comparing each variety to the wild type. Levels of variation within domesticated lines were not equivalent to those in the wild line (i.e., domesticated lines were composed of regions and accessions whereas wild lines were composed of populations and genotypes). Regardless, our goal was to test for effects of plant line (and in doing so, of domestication) while controlling for these levels of variation (as random effects) rather than attempting to explicitly compare these different levels of variation among domesticated and wild cabbages.

Because plant defences in induced plants results from the sum of pre-existing constitutive levels plus the induced response, defensive levels in the induced mode does not properly represent variation in inducibility, i.e. in the ability to increase defences after induction. We therefore used a bootstrapped approximation on our data as the best approach for replicating within-genotype variation in inducibility following *Moreira et al*.^45^, where we estimated inducibility of glucosinolate concentrations in each plant as the difference between the concentration of each induced plant and that of four control plants of the same genotype (concentrations of control plants represent constitutive levels). This resulted in four estimates of glucosinolate inducibility which were treated as repeated measures on the same subject^45^. This procedure also circumvented a spurious result arising when testing for a relationship between constitutive and inducible defences, as estimates of inducibility also include values of constitutive defences (i.e., response is contained in the predictor variable). To determine whether there was an effect of plant domestication on inducibility, we ran separate general linear mixed models for aliphatic and indole glucosinolates and in each case entered inducibility of each phytochemical class as a response variable and plant line, constitutive defences, and their interaction as explanatory variables. In addition, block, population/region nested within plant line, and maternal line/accession nested within population/region were included as random factors. The effect of constitutive defences in the model tested for an association between induced and constitutive defences, where a negative association suggests a trade-off between these defensive strategies. In addition, the interaction between plant line and constitutive defences tests whether this relationship differs among the plant lines. In addition, we also included leaf damage score weighed by leaf area as a covariate in both models. Finally, to test for trade-offs between constitutive and induced defences we also performed genetic correlations (using genotypic means) between constitutive glucosinolates and their inducibility.

We also investigated whether constitutive concentrations of glucosinolates differed between leaves and stems, and whether differences in defence investment among plant lines were greater for leaves than for stems. For this, we ran separate general linear mixed model for the concentration of indolic and aliphatic glucosinolate in control plants. Glucosinolate concentrations were entered as a response variable and plant line, tissue (leaf or stem), and their interaction as explanatory variables. Block, population/region nested within plant line and maternal line/accession nested within population/region were also included as random factors. In addition, we included “plant ID” as a random factor to account for repeated measures (one for each plant tissue) on each plant.

All analyses were performed with PROC MIXED in SAS 9.4 (SAS Institute, Cary, NC)^46^. When necessary, we log-transformed original variables to achieve normality of the residuals. We performed Tukey’s HSD (Honestly Significant Difference) post hoc ANOVA procedure to determine if there were differences between treatments. The formulation of the mixed models in SAS is shown in the Fig. SM1 of the Supplementary Material.

## Electronic supplementary material

Supplementary Material
