# Supplementary material for: Plant domestication decreases both constitutive and induced chemical defences by direct selection against defensive traits
Source: Sci Rep. 2018 Aug 23;8:12678. doi: 10.1038/s41598-018-31041-0 (PMC6107632; doi:10.1038/s41598-018-31041-0)
Supplement: Supplementary file 1 — Supplementary Material [file 41598_2018_31041_MOESM1_ESM.docx]

**Plant domestication decreases both constitutive and induced chemical defences by direct selection against defensive traits**

Xoaquín Moreira^1*^, Luis Abdala-Roberts^2^, Rieta Gols^3^, and Marta Francisco^1*^

^1^Misión Biológica de Galicia (MBG-CSIC), Apartado de correos 28, 36080 Pontevedra, Galicia, Spain.

^2^Departamento de Ecología Tropical, Campus de Ciencias Biológicas y Agropecuarias, Universidad Autónoma de Yucatán, Apartado Postal 4-116, Itzimná. 97000. Mérida, Yucatán, México.

^3^Laboratory of Entomology, Wageningen University, PO Box 16, 6700 AA, Wageningen, The Netherlands.

**Table SM1.** Information about accessions from the germplasm collection of Biological Mission of Galicia-CSIC (Spain) used in this study. We show the name of the accession, variety (*Brassica oleracea var. acephala or B. oleracea var. capitata*), region in Galicia (Cantabric, Atlantic or Interior) and the locality.

| **Accession** | **Variety** | **Region** | **Locality** |
| --- | --- | --- | --- |
| MBG-BRS0568 | *B. oleracea acephala* | Cantabric | Trasbar- Castelo- Cervo |
| MBG-BRS0566 | *B. oleracea acephala* | Cantabric | Valdemiros- SanPedro-Viveiro |
| MBG-BRS0477 | *B. oleracea acephala* | Cantabric | San Xoán, Moeche, Cedeira |
| MBG-BRS0351 | *B. oleracea acephala* | Cantabric | Xove |
| MBG-BRS0478 | *B. oleracea acephala* | Cantabric | A Barqueira, Cerdido |
| MBG-BRS0365 | *B. oleracea acephala* | Atlantic | Lérez, Pontevedra |
| MBG-BRS0366 | *B. oleracea acephala* | Atlantic | Moaña |
| MBG-BRS0045 | *B. oleracea acephala* | Atlantic | El Rosal |
| MBG-BRS0047 | *B. oleracea acephala* | Atlantic | Baredo, Bayona |
| MBG-BRS0043 | *B. oleracea acephala* | Atlantic | Mos |
| MBG-BRS0334 | *B. oleracea acephala* | Interior | Rozabales, Manzaneda |
| MBG-BRS0212 | *B. oleracea acephala* | Interior | Mourisca, Viana do Bolo |
| MBG-BRS0281 | *B. oleracea acephala* | Interior | A Rúa |
| MBG-BRS0335 | *B. oleracea acephala* | Interior | Larouco |
| MBG-BRS0151 | *B. oleracea acephala* | Interior | Viana do Bolo |
| MBG-BRS0569 | *B. oleracea capitata* | Cantabric | Trasbar-Castelo-Cervo |
| MBG-BRS0176 | *B. oleracea capitata* | Cantabric | Betanzos |
| MBG-BRS0693 | *B. oleracea capitata* | Cantabric | Cedeira |
| MBG-BRS0452 | *B. oleracea capitata* | Cantabric | San Mateo, Narón |
| MBG-BRS0402 | *B. oleracea capitata* | Cantabric | Betanzos |
| MBG-BRS0057 | *B. oleracea capitata* | Atlantic | Bueu |
| MBG-BRS0704 | *B. oleracea capitata* | Atlantic | Nogueira,Viso |
| MBG-BRS0737 | *B. oleracea capitata* | Atlantic | Vilar de Matos, Forcadela |
| MBG-BRS0751 | *B. oleracea capitata* | Atlantic | Gruncheiras, Coiro |
| MBG-BRS0740 | *B. oleracea capitata* | Atlantic | Casas, Gulans |
| MBG-BRS0152 | *B. oleracea capitata* | Interior | Mourisca, Viana do Bolo |
| MBG-BRS0639 | *B. oleracea capitata* | Interior | Petin |
| MBG-BRS0638 | *B. oleracea capitata* | Interior | Galicia (Carballeda de Valdeorras) |
| MBG-BRS0637 | *B. oleracea capitata* | Interior | Viana do Bolo |
| MBG-BRS0633 | *B. oleracea capitata* | Interior | Galicia (A Bola) |

**Figure SM1.** Example of a block of our split-plot design. The two herbivore treatments (two levels: control vs exposed to *Mamestra brassicae* larvae) were applied at the whole-plot factor and plant line (three levels: two domesticated varieties of *Brassica* *oleracea* [*B.* *oleracea* var. acephala and *B. oleracea* var. capitate] and their wild ancestor [*B. oleracea* spp. oleracea]) was the split factor. We randomly assigned the 15 genotypes of wild and domesticated lines within each split factor. Genotypes of wild cabbage consisted in five individual plants (i.e., maternal families composed of half-sibs) originating from three populations located within a range of 15 km on the southern coast of the United Kingdom. For both domesticated varieties, we used five accessions and each group of accessions belonged to one of three distinct regions located within a range of 270 km in Galicia (north-western Spain).

The formulation of the mixed model in SAS was:

PROC MIXED **data**=analysis covtest;

**class** block line region genotype ;

**model**  defence = line / outp=work.resid s;

**random** block region(line) genotype(region);

**lsmeans** line/diff ;

run;
